# Developing a General Population Screening Programme for Paediatric Type 1 Diabetes: Evidence from a Qualitative Study of the Perspectives and Attitudes of Parents

**DOI:** 10.1155/2024/9927027

**Published:** 2024-02-20

**Authors:** Lauren M. Quinn, Parth Narendran, Kirandeep Bhavra, Felicity Boardman, Sheila M. Greenfield, Matthew J. Randell, Ian Litchfield

**Affiliations:** ^1^Institute of Immunology and Immunotherapy, University of Birmingham, Birmingham B15 2TT, UK; ^2^Department of Diabetes, University Hospitals of Birmingham, Birmingham B15 2TH, UK; ^3^Sandwell and West Birmingham NHS Foundation, Birmingham B71 4HJ, UK; ^4^Division of Health Sciences, University of Warwick, Warwick CV4 7AL, UK; ^5^Institute of Applied Health Research, University of Birmingham, Birmingham B15 2TT, UK

## Abstract

**Introduction:**

With reliable tests and preventative treatments now available the United Kingdom has introduced a prototype population-based paediatric (ages 3–13) screening programme for type 1 diabetes (T1D). To aid its ethical and sustainable implementation this work explores parental views around the concept of this programme to determine how their involvement might be encouraged and supported. *Research Design and Methods*. Qualitative interviews were undertaken with 38 parents and the data were analysed using a purposely developed “Burden of Screening” framework, which presented the data within three domains describing the various elements of screening participation; pre-screening tasks designated to participants; factors influencing engagement with screening; and consequences of screening partici*pation*.

**Results:**

Regarding *pre-screening tasks designated to participants*, the importance of clear communication about the condition were apparent with parents expressing uncertainty of the benefits of screening against the potential anxiety engendered. In *factors influencing their engagement with screening* participants described their preference for less invasive testing techniques, the reassurance of structured support from healthcare professionals inherent within the programme, and the potential benefit of peer support. Regarding the *consequences of screening participation* parents described how a positive result might lead to overly protective behaviours, and anxiety from watching and waiting for the onset of symptomatic T1D.

**Conclusions:**

The benefits of T1D screening need to be clearly communicated to facilitate uptake. To this end the use of decision-support tools and better targeted educational materials should be explored. Post-testing, parents expressed preferences for peer support and access to psychological counselling.

## 1. Introduction

Type 1 diabetes (T1D) can be readily predicted by the presence of two or more islet-specific autoantibodies and dysglycaemia [1]. The benefits of its early prediction include education for symptom awareness, monitoring to track progression, a fivefold reduction in diabetic ketoacidosis (DKA) at onset, and improved glucose outcomes for the first years following diagnosis [2, 3]. Individuals at risk of T1D can also enter prevention trials, and more recently therapies have been licenced that can delay the development of symptomatic T1D [4, 5].

**Figure 1:**
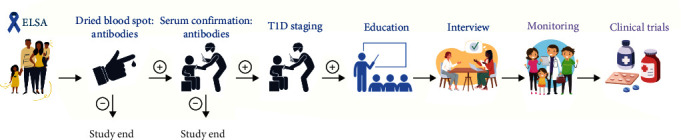
Summary of the ELSA screening programme [6].

With the rationale for a population-based screening programme for T1D established the UK's Early Surveillance for Autoimmune diabetes (ELSA) study is trialling a screening programme for children aged 3–13 years (Figure 1) [6]. Its eventual implementation across the United Kingdom requires approval by the UK National Screening Committee (UK NSC) [7] which involves consideration against four key ethical principles including the ability to improve health and well-being and the promotion of equality and diversity. Notably these principles also include “Treating People with Respect” which describes how screening programmes should understand the implications for the individuals involved, including the impact of their individual perspectives and values, and the potential consequences for screening participants and their families or carers [7].

Previous work exploring the social and ethical impact of screening participation in other conditions and diseases has revealed a number of common challenges [8]. These include the lack of understanding of the rationale for screening, the difficulties in complying with the requirements of the programme, and the management of the anxiety and fear engendered by a positive test [8–11]. The attitudes towards paediatric T1D screening in the UK are unknown, though it is likely that many of the acknowledged challenges and concerns around screening will emerge, the unpredictable lag time between a positive test and clinical onset of T1D is atypical of the conditions screened for in more established programmes [12]. Therefore, it is important that we understand the general population's perceptions towards paediatric screening for T1D and identify any unique challenges, barriers, and facilitators. To this end we undertook a qualitative exploration of the perceptions and perspectives of parents on the proposed screening programme. To enable a structured and transferable analysis of the data we have presented our findings within three pre-determined domains of a prototype “burden of screening” framework, specifically: information gathering and decision making; the factors affecting ongoing engagement with the programme; and the potential long-term impact of screening on the everyday lives of participants and their families.

## 2. Materials and Methods

### 2.1. Study Design

The study used a directed content analysis of qualitative data within the three domains of a purposely developed framework describing the burden placed on participants of screening, and their families and carers. The reporting followed the principles of Consolidated Criteria for Reporting Qualitative Studies (COREQ) [13] and the protocol describing the interview study from where the data were sourced (ELSA-1) has been published previously [6]. National research ethics approval was granted (IRAS: 294654).

### 2.2. The Burden of Screening Framework

An individual's engagement with any given healthcare intervention is a function of their ability to meet the requirements of the intervention, including its physiological and psychological impact [14]. In doing so they must also accommodate pre-existing and ongoing obligations to family, community, and work [14, 15]. Managing these sometimes contrasting requirements has been conceptualised as the “burden” placed on those participating [16] understood within three key domains; the work designated to an individual as a result of their participation, the factors that influence or exacerbate successful and ongoing participation, and finally, the consequences of participation for their everyday lives [16, 17]. We have adapted a pre-existing “Burden of Treatment” framework [18], utilising the same three domains but where previously the individual constructs were informed by the existing evidence on patient experiences of treatment they were informed by the literature exploring participants experience of screening to create a “Burden of Screening Framework.” The domains and constructs of the “Burden of Screening” (BoS) framework are described in Table 1.

### 2.3. Settings and Recruitment

Parents of all ages (with or without prior experience of diabetes) with children aged 3–13 years were eligible to take part from across England. They were invited to participate via either text message from their GP practice (*n* = 10), direct invitation from a member of the study team at community outreach events (*n* = 1 community hospital and *n* = 2 diabetes clinics), or advertisements placed on social media (Twitter and Facebook). Most parents were recruited by the text message from their GP (47%), followed by social media or snowball recruitment (32%), community events (16%), or via newsletter (5%). Parents were offered a £20 gift voucher for completing the interview.

Potential participants were provided with the information sheet and offered the opportunity to ask any questions before providing informed consent prior to the commencement of the interview. Those invited to interview were then purposively selected from those consented with the final sample representing various regions, ethnicity, occupation, parental age, and socioeconomic status according to the National Statistics Socioeconomic classification. Our intended sample size was 20–30 participants (though these may include two parents being interviewed simultaneously) in line with evidenced recommendations of the number capable of reaching data saturation [6, 31].

### 2.4. Data Collection

The interviews were semi-structured and were conducted by telephone, video-call, or face-to-face according to participant preference [32]. All interviews were conducted in 2022 and prior to each participant received either a verbal description of ELSA or a PowerPoint presentation describing the same to help frame the discussions (*Supplementary 1*: https://www.youtube.com/watch?v=-FriaK3cN4M). The topic guide (containing interview questions and prompts) was prepared using the existing literature on T1D screening and developed by the qualitative team (SMG, IL, FB, PN, LQ) and co-applicants including parents with a child with T1D. The topic guide asked participants to describe their understanding of T1D, the concept of screening, and included hypothetical questions around their child's participation including their reaction if their child tested positive. The topic guide was continually reviewed throughout the data collection period to ensure emerging topics were captured. The full topic guide can be found in *Supplementary 2*.

The majority of interviews (*n* = 30) was conducted by the first and last authors (LQ, clinical research fellow (female) and trained qualitative researcher, and IL (male) a senior qualitative researcher) with over 10 years' experience. Three interviews were conducted by three additional members of the research team also trained in qualitative research (PN, medical doctor (male), KB, medical doctor (female), and FS, medical student (female)) to support their ongoing development as qualitative researchers. The participants were not known to any of the interviewers. The interviews were transcribed by a third-party provider with whom there was a contractual confidentiality agreement and the data managed using N-Vivo v10 software.

### 2.5. Analysis

The data were analysed using a directed content analysis using an unconstrained matrix' approach suggested by Elo and Kyngäs allowed the development and inclusion of emergent themes within the established framework and meant that all the data were accommodated [33, 34]. First a selection of transcripts (*n* = 3) were independently analysed by four authors LQ, IL, SMG (Professor of Medical Sociology), and FB (Professor of Social Science). Following coding, we identified important parallels between the burden of chronic disease/burden of treatment [35] framework and the burden of screening. We henceforth used transcript data to develop the BoS framework and the qualitative team frequently met to discuss how to organise the data within the BoS framework before LQ analysed the remainder of the interviews in regular contact with the research team. The final allocation of data within the framework was consensually agreed by all authors. Throughout, we use rich verbatim accounts to illustrate the perceived burdens of screening prior, during and following participation in a proposed paediatric T1D screening programme (ELSA).

## 3. Results

In total, 129 participants expressed an interest in participation, 84 parents consented to take part but some were lost to follow-up and for some groups, e.g., families with a child/immediate family member with T1D we had reached data saturation and did not require further interviews. A final number of 34 interviews (38 parents in total) were completed and included in the analysis. The characteristics of participants are further summarised in Table 2. The interviews lasted between 37 and 87 min with a median duration of 54 min. Overall, 26 interviews were conducted by video-call, two of which had a translator present, three by telephone call, and two face-to-face both with a translator. The video presentation (https://www.youtube.com/watch?v=-FriaK3cN4M&t=4s) of the ELSA programme preceded 26 of the 34 interviews with the remainder receiving a verbal description of the programme from the researcher.

### 3.1. Qualitative Results

Below, we present the qualitative data within each domain and the related construct as defined within the BoS framework with exemplar quotes, chosen because they most clearly articulate a theme endorsed by multiple parents. The identifier following each quote corresponds to the participant code, whether mother or father, ethnicity and the number of children they have.

#### 3.1.1. Pre-Screening Tasks Designated to Participants

The parents we interviewed described their (mis) understanding of type 1 and type 2 diabetes, their perception of risk in the context of the rationale for screening and provided insight into the decision-making process they might adopt.

*(1) Understanding of Illness or Condition*. Individuals contemplating participation in any screening programme are expected to possess or accrue a basic understanding of the disease or conditions for which they are being screened [24]. In this instance a number of participants either conflated type 1 with type 2 diabetes or were concerned others would, as one mother commented:

“How do you get round the fact that already people don't really understand the difference between type 1 and type 2 - and so you screen as low risk of one thing whilst remaining high risk for another thing?” (Participant code P08, White British Mother, 1 child aged 6 years).

*(2) Rationale for Screening*. Parents consenting to their child joining any screening programme are expected to balance the impact of participation against the impact and potential risks involved [20–22]. For many of the parents we spoke to, particularly those without prior experience of diabetes, they felt the risk was so low as to be negligible compared to the potential anxiety that might result from participation. As one mother explained:

“…so, I have a son who is 11…if we were told he had to go for a ‘diabetes test', or a ‘screening test' - I know he is going to have 1001 questions for me. He is going to be very anxious about it as well, and it might be worrying for him. So, I am thinking ‘I don't know? Since we're saying that the prevalence is quite low, I just don't know if it's worth putting them through all that?' …” (P01, Black African Caribbean Mother, 3 children aged 8–13 years).

*(3) Decision-Making*. Though, it is expected that in ELSA parents will make participation decisions on behalf of younger children, those with older children felt it was important that they should contribute to the decision-making process:

“It would be entirely up to him if he wanted to do that [participate]. If he would feel empowered by doing that and it would help him then yes…but it would certainly be a decision that is made with him, and we would let him lead on that situation.” (P028, White British Mother, 1 child aged 8 years).

#### 3.1.2. Factors Influencing Engagement with Screening

Participants described their considerations of the time and resource necessary to comply with the screening programme's requirements, the impact of their personal experience of diabetes, and the potential value of structured clinical support being incorporated in the programme.

*(1) Managing Tasks Involved in Screening*. In considering the testing and monitoring processes that would be associated with ELSA, parents expressed a preference for simple, minimally invasive tests that could be conducted in their own home:

“I don't think I have much of a problem with having to have a little prick or the venous blood sample taken, because it will just cause some distress for that couple of minutes or whatever and then it's over.” (P01, Black African Caribbean Mother, 3 children aged 8–13 years).

The ELSA programme dictates that the monitoring process following a positive test requires repeated tests and consultations which was a concern for some parents:

“[what] made me nervous was about them having to take blood tests then every two months, and it's like ‘Oh man, that's pretty heavy duty for a kid who doesn't have it yet!” (P028, White British Mother, 1 child aged 8 years).

*(2) Personal Characteristics*. Prior experience of diabetes influenced the level of interest in screening. For example, one mother explained how her personal understanding of gestational diabetes meant she would be more inclined to support participation:

“I think the screening for me definitely I would want to do anyway because of my own experience of having gestational diabetes and [child] being at greater risk potentially of type 1 or type 2 diabetes,” (P021, White British Mother, 2 children, 1 child aged 6 years).

The individual's more general attitude to health and well-being also appeared to impact their likelihood of participation in ELSA. For example, one mother we spoke to reflected on how her trust in NHS brokered care initiatives had been reduced due to what they perceived as the miscommunication of risk during the COVID pandemic:

“…because I feel that risk has been misappropriated over the last two years, feel very passionately about that,… I think we have introduced a set of worries to our children that didn't exist before,… and a way that we talk about disease that isn't healthy…if you'd asked me in 2019 I would have said probably “Yes!” - with very little doubt. Whereas now - with the current culture being as it is…? I feel…we are overstating risk all of the time…” (P08, White British Mother, 1 child aged 6 years).

Another parent described how their general anxiety about health and well-being meant they would view ELSA as a valuable “health check”:

“I would say I'm an anxious parent…I do worry about more things,… like I've literally just said to my husband I'm concerned that my little boy has drank an awful lot today, because he's drank two litres of squash…If I could have a full body MOT every year I think I would, I'm that type of person!” (P027, White British Mother, 1 child aged 5 years).

*(3) Structural Factors*. In thinking about the consequences of their child testing positive, parents described the reassurance of knowing they had ready access to reliable clinical advice on how to manage their child's health:

“it's hard isn't it to know as parents that your child could develop something….so if you've got healthcare professionals along the way to support you - and to guide you -it eases that doesn't it? It eases the stress of knowing that your child might get diabetes.” (P05, White British Mother, 3 children, 1 child aged 6 years).

Similarly, parents described the potential benefit of the provision of psychological support inherent in the ELSA programme:

“…psychological support…where some child psychologist can then discuss with them how they feel and that sort of thing…and depending on their age, they want to be a bit brave, when in fact they are not feeling like it.” (P010, White British Mother, 2 children aged 10 and 12 years).

Related to this, several parents felt peer support might also be of benefit for them as parents following a positive test, enabling them to learn from others in a similar situation including those with a child that is living with T1D:

“Perhaps talking to other families that have children with diabetes themselves, that would be useful…if they've got any hints or tips, or things that they do so they don't forget things, or things that the children can help with that live with it themselves,” (P022, White British Mother, 2 children aged 6 and 10 years).

There was also the hope expressed that peer support might be developed specifically for their child to help them deal with the situation. As one parent explained:

“I suppose almost like some social time or some group time where young people can chat to each other, and where it's not necessarily to talk about diabetes, but just have a little bit of a playgroup or a youth group where they can just be together and it's normalised,” (P07, White British Mother, 2 children, 1 aged 6 years).

#### 3.1.3. Consequences of Screening

Parents described concerns over the possible financial consequences of participating in ELSA, the effect it might have on their child's daily activities, the potential emotional impact of a positive test on them and their child, and finally the way in which they might accommodate the results of a positive test in their everyday lives:

*(1) Financial Impact*. There can be a range of financial impacts resulting from involvement in a screening programme that produces a positive result [29]. For example, one parent expressed concern that it might increase the cost of financial services such as health insurance:

“You start to think…‘Does it have implications for medical insurance and all things like that?'… you think… ‘How much does that information affect the rest of your life when you don't necessarily have the condition but you're at risk from getting the condition?'” (P02, White British Mother, 2 children aged 4–6 years).

*(2) Impact on Professional, Social, Family Life, and Leisure Activities*. In describing the potential implications of a positive test on their child's daily lives, parents described how they might become more protective due to perceptions of their child's vulnerability. This led some to consider how they might minimise that risk, for example by home schooling:

“If the condition is critical or chronic condition, I think we have to withdraw him from school so that I can take care of him at home, teach him at home.” (P014, Multiple ethnic groups Father, 2 children, 1 aged 4 years).

*(3) Emotional Impact*. Parents described the anxiety they would feel following a positive test due to not knowing when symptomatic T1D would present:

“…it would feel like a ticking timebomb…I don't know how I'd cope with that… Am I just staring at them? Looking for them drinking more? Going to the toilet more?… just living on the edge, constantly wondering when that tipping point is going to come,” (P02, White British Mother, 2 children aged 4–6 years).

*(4) Living with the Outcome*. Parents who have friends or relatives with children diagnosed with T1D following DKA felt that a positive result via ELSA would reduce the trauma of diagnosis and potentially allow them to better to prepare for its onset. As one mother explained:

“But a lot of people I know who have it…first found out [via DKA], and they were in the hospital for weeks. I would just not want that to happen to my son, that all of a sudden it comes crashing down. It's better to know like ‘Okay this could happen, let's prepare for it' – give you time to adjust to this situation.” (P028, White British Mother, 1 child aged 8 years).

## 4. Discussion

### 4.1. General Findings

This is the first qualitative study to explore general population views towards autoantibody screening for T1D in the United Kingdom. The use of the BoS framework has provided a systematic means of considering the perspectives of parents by deconstructing participation in screening within the three domains. In the first, the *pre-screening tasks designated to participants* the importance of clear communication about the condition were apparent with the re-emergence of the common conflation of type 1 and type 2 diabetes. There was also uncertainty about the benefits of participation against the anxiety that might ensue; parents were comfortable making the decision to participate for younger children but felt older offspring would ideally be involved. In considering the impact of the second domain, *factors influencing their engagement with screening*, participants described their preference for less invasive testing techniques, how the impact of their personal experience of diabetes would motivate participation, and the reassurance of knowing that there was structured clinical support associated with the programme if a positive test resulted. Regarding the third domain, the possible *consequences of screening participation* parents described fears of an increase in health insurance premiums, how a positive result might lead to overly protective behaviours among the anxiety of waiting for the onset of symptomatic T1D. Meanwhile those with experience of diagnosis via DKA felt a positive result would lessen the impact of the diagnosis and enable preparedness.

### 4.2. Specific Findings

#### 4.2.1. Pre-Screening Tasks Designated to Participants

The common conflation of type 1 and type 2 diabetes that emerged [36] might serve to decrease participation in populations where stigma or blame is attached to any “diabetes” but also influence participation of those that mistakenly believe onset of T1D can be prevented by lifestyle change [36]. This again highlights the importance of clear messaging around the condition being screened [24, 37, 38], which is also a requirement of ethically informed participation [24] and has proven valuable in compensating for different levels of health literacy and increasing participation rates among underserved populations [39]. To help participants better conceptualise risk (presented in the ELSA information as 3 in 1,000) and balance benefits against potential harms of participation, other screening programmes have utilised a range of decision tools to support recruitment including brochures, audio–visual materials, educational sessions and interactive websites, all of which might be usefully trialled with future iterations of the ELSA programme [25].

#### 4.2.2. Factors Influencing Engagement with Screening

The impact of screening participation on both temporal and financial resources described by our participants have been recognised previously by the UK's Department of Health and Social Care, in particular their disproportionate impact on underserved groups [40]. To overcome this potential deterrent to participation a number of recommendations have emerged including targeted financial incentives and recruitment campaigns that directly address structural barriers to participation [41].

Parental motivation for their child's potential participation in ELSA was drawn from their previous experience of the condition, as observed in other screening programmes [38]. Another factor favouring participation was the assurance of regular contact with healthcare professionals through the ELSA programme, reflecting the sentiments expressed by the participants of other screening programmes [42–46].

#### 4.2.3. Consequences of Screening Participation

Parents described the financial implications of a positive result; economic concerns which have materialised in other screening programmes [44, 45]. Parents also described the desire to protect their child if they tested positive and suggested risk averse behaviours (symptom monitoring, healthier lifestyle) observed previously in parents with children genetically predisposed to developing T1D [46]. Similarly, these positive effects have been observed in other screening programmes where involvement raised awareness of healthy behaviours for the wider family [47].

A significant consequence of screening was the anxiety some parents experienced waiting for the onset of T1D as witnessed previously in work exploring genetic risk of T1D where it was particularly pronounced among mothers [46, 48]. However, those parents aware of diagnosis via DKA saw the benefit of being forewarned of the eventual onset of symptomatic T1D. This reflects the little evidence that exists from genetic screening programmes for T1D which indicates that the “soft landing” provided by prior knowledge can reduce parental stress compared to families diagnosed outside of screening [49]. Similarly, positive effects have been observed in other screening programmes where participation raised awareness of healthy behaviours for the entire family [47].

#### 4.2.4. Strengths and Limitations

Describing the issues and challenges of screening within the structured and clearly defined BoS framework proved a useful means of aiding the further development of ELSA but also paediatric screening programmes for the other conditions. The use of an *a priori* framework can be considered a constraint on the interpretation of the data and although the topic guides were not specifically designed to populate the BoS framework its comprehensive nature meant all of the data could be accommodated within the BoS domains and constructs. That we did not uncover any data relating to “structural changes” is more likely due to the hypothetical nature of the discussion.

The rigour of the study and its reporting was upheld throughout by employing a number of recommended strategies including sharing clear and accurate records of the analysis across the team, the explicit description of the experience and prior knowledge of the interviewers, and by using rich and verbatim descriptions of participants' comments.

Although, we recruited a wide range of participants in an attempt to create a representative sample we acknowledge they had “opted-in” to the qualitative study which might have led to a disproportionate number of participants with prior experience of diabetes and their being more receptive to the concept of screening than others. Offering a voucher for participation aided recruitment but may have introduced bias to the sampled population.

### 4.3. Conclusions/Implications for Practice

Although, the benefits of early recognition of T1D are clinically apparent, these need to be clearly communicated to support participation in T1D screening programmes. Perhaps unsurprisingly parents with previous experience of T1D demonstrated a greater understanding of the benefits, the use of decision-support tools and targeted educational materials might still be considered to better communicate the benefits (and risks) of participation for all potential participants and their families. There is also a need for further consideration of the support offered to address the anxiety described by some participants and their families reflecting their preferences for psychological interventions and structured connections to peers. Both supportive approaches warrant further research to understand how they can be optimised to help children and families come to terms with a positive result and support informed participation.

## Figures and Tables

**Table 1:** Burden of screening framework.

Domain	Construct	Definition
Pre-screening tasks designated to participants	Understanding of target disease or condition	Acquiring, understanding, and interpreting information from a number of sources (including specifically developed materials, the internet, and social and mainstream media) on the causes, symptoms, available treatments, prognosis and long-term management of the target disease(s) or condition(s) [19]
	Rationale for screening	The process of acquiring knowledge of the benefits of screening vs associated risk, including both targeted, and general population screening [20–22]
	Decision-making	The autonomous decision to participate informed by their perception of the benefits and risks of participation [23, 24] often supported by purposely designed decision tools [25]

Factors influencing engagement with screening	Managing tasks involved in screening	The impact of performing the tasks related to the screening process. Includes administrative tasks involved in managing and maintaining the screening process, precautions taken before or when performing tests, and planning and fulfilling visits to the appropriate location/healthcare provider [26]
	Personal characteristics	Personal or first-hand experience of illness/condition (including first degree relative); level of health literacy; individual beliefs around health and well-being [27, 28]
	Situational factors	The contextual factors that determine whether and how patients can manage the processes involved in screening including changes in personal circumstances (such as a change in job) or in personnel of their healthcare team [8]
	Structural factors	The nature and accessibility by participants of resources directly related to the screening process. Includes continued contact or access to healthcare professionals, structured support and education mechanisms and peer support [8]

Consequences of screening participation	Impact on professional, social, family life, and leisure activities	The professional and social consequences that affect participants as a result of the screening process and/or the results. This includes balancing the pursuit of meaningful activities with appropriate attention to (potential) illness needs, and the opportunity cost in professional/social life [8]
	Financial impact of healthcare tasks designated to participants post-screening	Direct costs of (actual/potential) follow-up related to the value of resources used in supporting adherence to the screening programme, or the results of screening. Also includes Indirect costs of screening such as time lost from work [29]
	Emotional impact of screening participation	The varying ability of participants to explore, express and process various emotions in response to the (potential) loss of health or functioning resulting from participation [20]
		Includes intrinsic factors relating to resilience and inner resources, and external factors relating to the availability of social support (e.g., as drawn from family, friends, community, or spiritual beliefs) [20]
	Living with the outcome	The capability, preparedness, and extent to which an individual can or will accommodate the results of participation. This includes the ability to understand and manage recommendations from healthcare providers [30]

**Table 2:** Characteristics of parent participants.

*Mother or father*				
Mother			Father	
24			10	
*Age of parent in years ^*∗*^*				
25–29	30–34	35–39	40–44	45+
5	4	12	8	3
*Number of children*				
1	2		3	4+
8	18		5	3
*Parental ethnicity*				
Afro-caribbean	Arabic	Asian	Mixed/multiple	White British
5	6	2	4	20
*Location by region ^*∗∗*^*				
North-East	North-West	Midlands	South-East	South-West
2	1	28	2	2
Socio-economic Status—National Statistics Socioeconomic Classification (NS-SEC)				
1–2 Higher/lower managerial, professional occupations	3–4 Intermediate occupations, small employers	5–6 Lower supervisory, semi-routine	7–8 Routine occupations, never worked/unemployed	Unknown or not classified
22	5	1	7	3
Experience of diabetes ^*∗∗∗*^				
Parent with T1D	Child with T1D	Other family member/friend with T1D	Family member with T2D	No family history
3 (Mothers)	7	4	4	17

^*∗*^Unknown for two;  ^*∗∗*^unknown for three;  ^*∗∗∗*^unknown type of diabetes for three.

## Data Availability

The data are not publicly available as it contains information that could compromise the privacy of research participants.

## Supplementary Materials

Supplementary 1 Summary of ELSA programme for participants.

Supplementary 2 Summary of topic guide for participants.

## References

[1] Insel R. A., Dunne J. L., Atkinson M. A. (2015). Staging presymptomatic type 1 diabetes: a scientific statement of JDRF, the Endocrine Society, and the American Diabetes Association. *Diabetes Care*.

[2] Lundgren M., Jonsdottir B., Larsson H. E., DiPis Study Group (2019). Effect of screening for type 1 diabetes on early metabolic control: the DiPiS study. *Diabetologia*.

[3] Ziegler A.-G., Kick K., Bonifacio E. (2020). Yield of a public health screening of children for islet autoantibodies in Bavaria, Germany. *JAMA*.

[4] Perdigoto A. L., Preston-Hurlburt P., Clark P. (2019). Treatment of type 1 diabetes with teplizumab: clinical and immunological follow-up after 7 years from diagnosis. *Diabetologia*.

[5] FDA (2022). FDA approves first drug that can delay onset of type 1 diabetes. https://www.fda.gov/news-events/press-announcements/fda-approves-first-drug-can-delay-onset-type-1-diabetes.

[6] Quinn L. M., Shukla D., Greenfield S. M. (2022). EarLy Surveillance for Autoimmune diabetes: protocol for a qualitative study of general population and stakeholder perspectives on screening for type 1 diabetes in the UK (ELSA 1). *BMJ Open Diabetes Research & Care*.

[7] N. S. Committee (29 September 2022). Criteria for a population screening programme. https://www.gov.uk/government/publications/evidence-review-criteria-national-screening-programmes/criteria-for-appraising-the-viability-effectiveness-and-appropriateness-of-a-screening-programme.

[8] Camilloni L., Ferroni E., Cendales B. J. (2013). Methods to increase participation in organised screening programs: a systematic review. *BMC Public Health*.

[9] Duffy S. W., Myles J. P., Maroni R., Mohammad A. (2017). Rapid review of evaluation of interventions to improve participation in cancer screening services medical. *Journal of Medical Screening*.

[10] Baldwin D. R., Brain K., Quaife S. (2021). Participation in lung cancer screening. *Translational Lung Cancer Research*.

[11] Wardle J., Pope R. (1992). The psychological costs of screening for cancer. *Journal of Psychosomatic Research*.

[12] Sims E. K., Besser R. E. J., Dayan C. (2022). Screening for type 1 diabetes in the general population: a status report and perspective. *Diabetes*.

[13] Booth A., Hannes K., Harden A., Noyes J., Harris J., Tong A., Moher D., Altman D. G., Schulz K. F., Simera I., Wager E. (2014). COREQ (Consolidated Criteria for Reporting Qualitative Studies). *Guidelines for Reporting Health Research: A User’s Manual*.

[14] Eton D. T., Yost K. J., Lai J. S. (2017). Development and validation of the patient experience with treatment and self-management (PETS): a patient-reported measure of treatment burden. *Quality of Life Research*.

[15] Ridgeway J. L., Egginton J. S., Tiedje K. (2014). Factors that lessen the burden of treatment in complex patients with chronic conditions: a qualitative study. *Patient Preference and Adherence*.

[16] May C. R., Eton D. T., Boehmer K. (2014). Rethinking the patient: using burden of treatment theory to understand the changing dynamics of illness. *BMC Health Services Research*.

[17] Spencer-Bonilla G., Quiñones A. R., Montori V. M., International Minimally Disruptive Medicine Workgroup (2017). Assessing the burden of treatment. *Journal of General Internal Medicine*.

[18] Litchfield I., Calvert M. J., Kinsella F., Sungum N., Aiyegbusi O. L. (2023). “I just wanted to speak to someone- and there was no one026;”: using burden of treatment theory to understand the impact of a novel ATMP on early recipients. *Orphanet Journal of Rare Diseases*.

[19] McMullan M. (2006). Patients using the Internet to obtain health information: how this affects the patient–health professional relationship. *Patient Education and Counseling*.

[20] Collins R. E., Lopez L. M., Marteau T. M. (2011). Emotional impact of screening: a systematic review and meta-analysis. *BMC Public Health*.

[21] Deutekom M., Vansenne F., McCaffery K., Essink-Bot M.-L., Stronks K., Bossuyt P. M. M. (2011). The effects of screening on health behaviour: a summary of the results of randomized controlled trials. *Journal of Public Health*.

[22] Vernon S. W. (1999). Risk perception and risk communication for cancer screening behaviors: a review. *JNCI Monographs*.

[23] Irwig L., McCaffery K., Salkeld G., Bossuyt P. (2006). Informed choice for screening: implications for evaluation. *BMJ*.

[24] NHS (August 2022). NHS screening 2021. https://www.nhs.uk/conditions/nhs-screening/.

[25] Stacey D., Légaré F., Lewis K. (2017). Decision aids for people facing health treatment or screening decisions. *Cochrane Database of Systematic Reviews Review*.

[26] Dressler J., Johnsen A. T., Madsen L. J., Rasmussen M., Jorgensen L. N. (2021). Factors affecting patient adherence to publicly funded colorectal cancer screening programmes: a systematic review. *Public Health*.

[27] Hann K. E. J., Freeman M., Fraser L. (2017). Awareness, knowledge, perceptions, and attitudes towards genetic testing for cancer risk among ethnic minority groups: a systematic review. *BMC Public Health*.

[28] Vallone F., Lemmo D., Martino M. L. (2022). Factors promoting breast, cervical and colorectal cancer screenings participation: a systematic review. *Psycho-Oncology*.

[29] De Klerk C. M., Gupta S., Dekker E., Essink-Bot M. L. (2018). Socioeconomic and ethnic inequities within organised colorectal cancer screening programmes worldwide. *Gut*.

[30] Johnson F., Ulph F., MacLeod R., Southern K. W. (2022). Receiving results of uncertain clinical relevance from population genetic screening: systematic review & meta-synthesis of qualitative research. *European Journal of Human Genetics*.

[31] Morse J. M. (2000). Determining sample size. *Qualitative Health Research*.

[32] DeJonckheere M., Vaughn L. M. (2019). Semistructured interviewing in primary care research: a balance of relationship and rigour. *Family Medicine and Community Health*.

[33] Bingham A. J., Witkowsky P., Vanover C., Mihas P., Saldaña J. (2022). Deductive and inductive approaches to qualitative data analysis. *Analyzing and Interpreting Qualitative Data: After the Interview*.

[34] Elo S., Kyngäs H. (2008). The qualitative content analysis process. *Journal of Advanced Nursing*.

[35] Tran V.-T., Barnes C., Montori V. M., Falissard B., Ravaud P. (2015). Taxonomy of the burden of treatment: a multi-country web-based qualitative study of patients with chronic conditions. *BMC Medicine*.

[36] Marciano L., Camerini A.-L., Schulz P. J. (2019). The role of health literacy in diabetes knowledge, self-care, and glycemic control: a meta-analysis. *Journal of General Internal Medicine*.

[37] O’Donovan B., Mooney T., Rimmer B. (2021). Advancing understanding of influences on cervical screening (non)-participation among younger and older women: a qualitative study using the theoretical domains framework and the COM-B model. *Health Expectations*.

[38] Young B., Bedford L., Kendrick D., Vedhara K., Robertson J. F. R., das Nair R. (2018). Factors influencing the decision to attend screening for cancer in the UK: a meta-ethnography of qualitative research. *Journal of Public Health*.

[39] Fuzzell L. N., Perkins R. B., Christy S. M., Lake P. W., Vadaparampil S. T. (2021). Cervical cancer screening in the United States: challenges and potential solutions for underscreened groups.” Preventive. *Preventive Medicine*.

[40] Reviews of Modern Physics (2022). Population screening: review of interventions to improve participation among underserved groups. https://www.gov.uk/government/publications/population-screening-improving-participation-in-underserved-groups/population-screening-review-of-interventions-to-improve-participation-among-underserved-groups.

[41] Mauro M., Rotundo G., Giancotti M. (2019). Effect of financial incentives on breast, cervical and colorectal cancer screening delivery rates: results from a systematic literature. *Health Policy*.

[42] Green B. B., Wang C. Y., Horner K. (2010). Systems of support to increase colorectal cancer screening and follow-up rates (SOS): design, challenges, and baseline characteristics of trial participants. *Contemporary Clinical Trials*.

[43] Mbah O., Ford J. G., Qiu M. (2015). Mobilizing social support networks to improve cancer screening: the COACH randomized controlled trial study design.. *BMC Cancer*.

[44] Le Bonniec A., Sun S., Andrin A., Dima A. L., Letrilliart L. (2022). Barriers and facilitators to participation in health screening: an umbrella review across conditions. *Prevention Science*.

[45] McQueen M. J. (2002). Some ethical and design challenges of screening programs and screening tests. *Clinica Chimica Acta*.

[46] Kerruish N. J., Healey D. M., Gray A. R. (2017). Psychosocial effects in parents and children 12 years after newborn genetic screening for type 1 diabetes. *European Journal of Human Genetics*.

[47] van der Aalst C. M., van Klaveren R. J., de Koning H. J. (2010). Does participation to screening unintentionally influence lifestyle behaviour and thus lifestyle-related morbidity?. *Best Practice & Research Clinical*.

[48] Smith L. B., Lynch K. F., Baxter J. (2014). Factors associated with maternal-reported actions to prevent type 1 diabetes in the first year of the TEDDY study. *Diabetes Care*.

[49] Smith L. B., Liu X., Johnson S. B. (2018). Family adjustment to diabetes diagnosis in children: can participation in a study on type 1 diabetes genetic risk be helpful?. *Pediatric Diabetes*.

