# Supplementary material for: Developing a General Population Screening Programme for Paediatric Type 1 Diabetes: Evidence from a Qualitative Study of the Perspectives and Attitudes of Parents
Source: Pediatr Diabetes. 2024 Feb 20;2024:9927027. doi: 10.1155/2024/9927027 (PMC12017103; doi:10.1155/2024/9927027)

**Supplementary file 1 – Pre-interview educational material**

**Qualitative interview: education script:**

1. The ELSA study is screening children to find out their risk of getting type 1 diabetes.
2. This short video will tell you about what type 1 diabetes is and what our screening programme involves.
3. Type 1 diabetes is an autoimmune condition, that is more common in children
4. This differs from type 2 diabetes which is caused by genetic and lifestyle factors and more common as we get older.
5. The pancreas is an organ in our tummy that produces insulin. Insulin is the key needed to unlock nutrition from our food.
6. In type 1 diabetes, the body’s own immune system attacks the pancreas. Antibodies, shown here contribute to this process.
7. Children with type 1 diabetes cannot produce their own insulin. Without insulin, children become very unwell and need to take replacement insulin straight away and continue this for the rest of their lives.
8. In the ELSA study, we are testing for these antibodies, because antibodies are markers of risk.
9. As the number of antibodies a child has rises, this increases their risk of getting type 1 diabetes.
10. For example, for children who have two of these antibodies, within the next 10 years, 3 in 4 of these children will develop type 1 diabetes.
11. In the general population, 3 in 1000 children are at high risk of getting type 1 diabetes, but we can only find these children by screening.
12. Children aged from 3 up to 13 years can take part in our screening programme.
13. So what does our screening programme involve?
14. Firstly, children will have a finger prick test, at home or in the community.
15. If this shows the child does not have antibodies, the child will not need any further follow-up in ELSA. This will be the case for over 99% of the children screened in our study.
16. If this shows the child has antibodies, we will need to do a blood test to confirm this.
17. If this test confirms antibodies, the child is at high risk and needs more blood tests to see how close to getting type 1 diabetes they are.
18. All children at risk in our study and their families, will be invited to an education session, to understand the symptoms to look out for in the future. We will also let families know about monitoring and research studies their child may be eligible for.
19. So, what needs to be considered?
20. We understand finding out a child is at risk is potentially distressing, but the ELSA study team will support the family and talk through the options available.
21. No screening test is 100% accurate, but we will use the best available tests.
22. Currently, there is no approved treatment to prevent type 1 diabetes.
23. So, what are the benefits of screening?
24. The earlier we screen, gives us more opportunity to intervene.
25. This means we can monitor high risk children more closely.
26. This might mean a blood test every few months, to check for antibodies over time and to identify sooner when children need to start insulin. This is before they get unwell and need to go into hospital.
27. Importantly, therapies are being trialled for children which aim to prevent or delay the start of T1D.
28. And only by screening, can we identify the children who could benefit from these research studies. Thank you for listening.

**Qualitative interview: presentation slides**


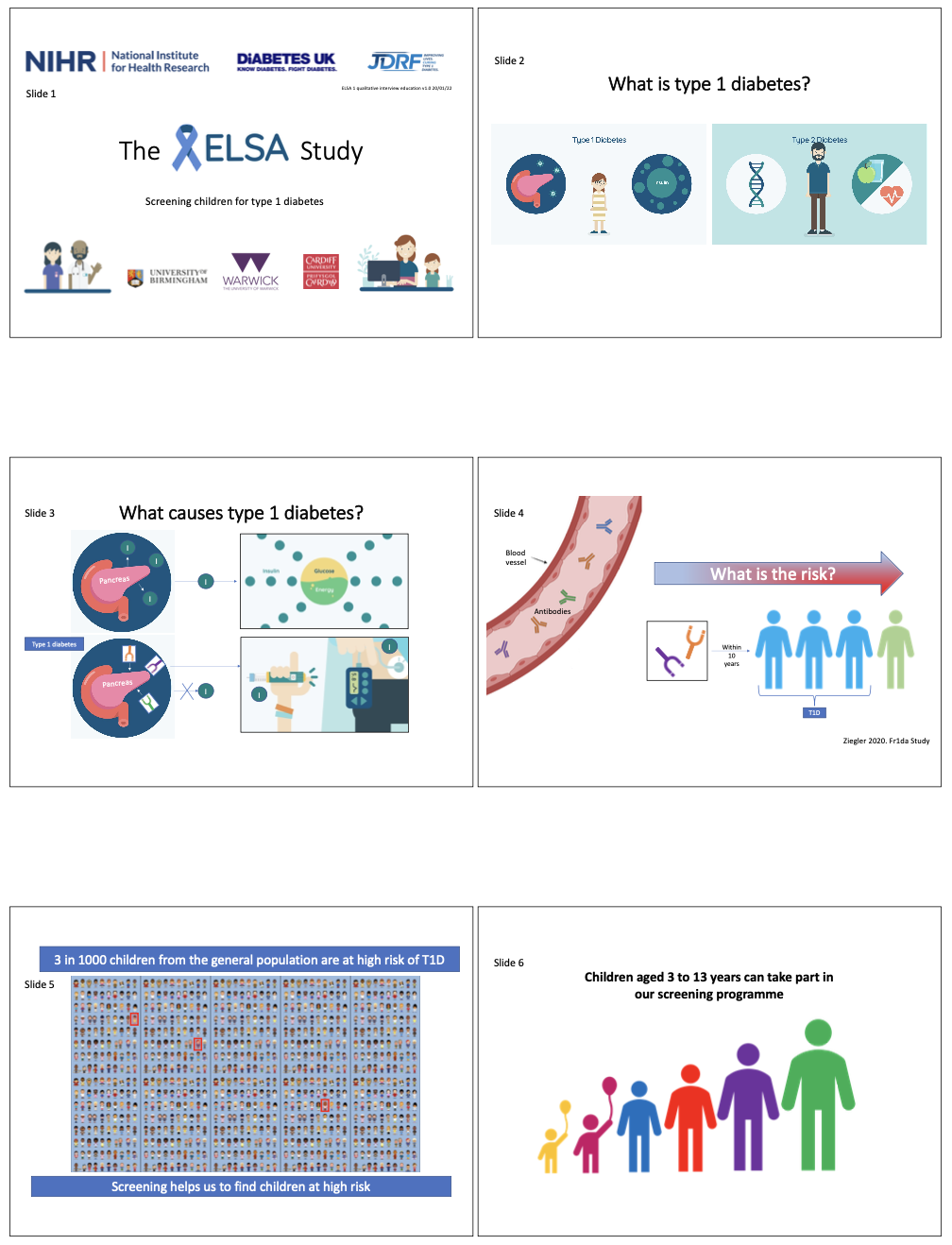


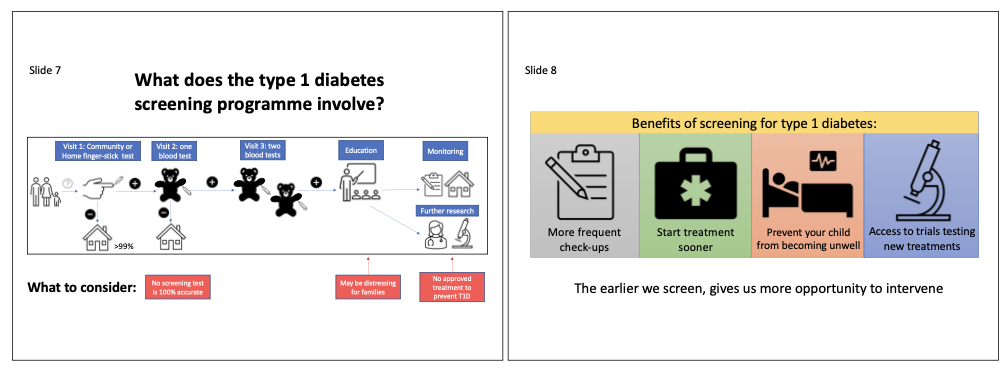

Supplement: Supplementary 1 — Summary of ELSA programme for participants. [file 9927027.f1.docx]
