# Supplementary material for: Developing a General Population Screening Programme for Paediatric Type 1 Diabetes: Evidence from a Qualitative Study of the Perspectives and Attitudes of Parents
Source: Pediatr Diabetes. 2024 Feb 20;2024:9927027. doi: 10.1155/2024/9927027 (PMC12017103; doi:10.1155/2024/9927027)
Supplement: Supplementary 2 — Summary of topic guide for participants. [file 9927027.f2.docx]

**Supplementary File 2 – Topic guide**

| **Question** | **Sub-question** | **Prompts** |
| --- | --- | --- |
| 1. What are your initial thoughts following the video presentation? 2. Do you have any questions you would like to ask? | *Corrections if required:*   - *Type 1 diabetes is a lifelong condition that cannot be cured.* - *The symptoms of type 1 diabetes include: thirst, increased urinary frequency, weight loss, and fatigue.* - *Type 1 diabetes is treated with insulin therapy.* - *Lifestyle measures are important in the management of type 1 diabetes, but alone are insufficient to manage the condition.* - *In a person with type 1 diabetes, without insulin therapy, the person will become very sick, and is at risk of diabetic ketoacidosis. In the long-term without insulin, the person would not survive.* | |
| 1. **Understanding of type 1 diabetes** 2. What do you understand by type 1 diabetes?      1. Were you aware of type 1 diabetes before this presentation?      1. Do you know of anyone who has type 1 diabetes (e.g. family, friends or colleagues)?      1. What do you know about how it affects their life? (what might a person with diabetes experience in their daily lives? | What do you understand about daily testing, medication, lifestyle considerations? | Positive, negative |
| **B) Views about screening for type 1 diabetes**   1. What are your first thoughts and feelings about the screening programme described in the presentation?      1. What are the reasons you can think of why a parent/guardian may not want to have their child screened?      1. Which parts make the screening programme appealing to parents? 2. Antibody negative 3. Antibody single or double positive 4. Thinking about your own family situation, is this something you would want your child to have?      1. Are there any reservations/concerns you would have about entering your child into this screening programme?      1. High risk children identified by screening, could take part in trials testing new treatments to delay the start or try to prevent type 1 diabetes. What are your thoughts and feelings about this? 2. If there was an effective treatment for prevention of T1D in children, how would this affect your thoughts and feelings about childhood screening? | [A] Imagine you have a little girl called [insert name e.g. Jasmine], who is 3 years old. You decide to do the type 1 diabetes screening test at the GP practice, when she has her Measles Mumps Rubella (MMR) vaccination. You receive a text message a week later, to say the result is negative. This means she is at low risk of getting type 1 diabetes in the future.  What would this make you think and feel?  [B] Imagine you have a child called [insert name e.g. Arun], who is 13 years old. Arun finds out about the screening programme at school and wants to take part. He discusses this with you and you decide to proceed. Arun has the screening test at school. A week later, you receive a text message to say Arun’s result is positive and that he needs some more blood tests to confirm this finding.  What would this make you think and feel?   1. Why or why not? 2. Age of your child, child’s personality, comorbidities, your own attitudes around health in general, religious beliefs 3. What do you consider to be the risks of trials? 4. Does knowing that there is no current treatment that can prevent or delay the start of type 1 diabetes change your views about whether we should screen? | Anxiety, time, cost, burden  Education, monitoring, research  Any differences from above?  -Fears/anxieties, age of your child, child’s personality, comorbidities, your own attitudes around health in general, religious beliefs  Positive, negative |
| **C) Practicalities and Mechanics of the study**   1. What do you think about what the screening process involves?      1. Where would you want to have the screening test done?      1. *What type of information would you want to be given in order to help you make a decision about whether to take part in a screening programme?*      1. How would you want the results to be communicated to you?      1. If your child was at risk of type 1 diabetes, what education would you want?      1. What are your thoughts on an opt out screening programme for children, meaning all children would have the screening test routinely, unless the parent/child chose not to have it?      1. On balance, would you have your child screened to find out their risk of type 1 diabetes? | 1. What are your reactions to the: finger prick test, confirmatory venous test, oral glucose tolerance test/fasting, education session 2. How do you think your child would cope/tolerate each test?   *Was there enough, easily understandable, anything else you would like to know?*  Within what time frame would you expect to receive the results: days/weeks/months   - *What did you think about the information on the website?*   How soon after finding out the test result – days, weeks?  a. What factors would influence your decision  b. How would the following affect your decision: accuracy of the test/ preventative treatment approved  Why or Why not? | Thoughts and feelings on the screening programme; burden; ethics; intervention coherence; opportunity costs; perceived effectiveness; self-efficacy  GP surgery/Childhood vaccination programme e.g. MMR vaccination/School/Local community centre/Home testing/Any other?  *Written/Website/Face-to-face*   - Remotely or in person - Letter/e-mail/text message/phone call - By a member of the research team/ your GP/ other healthcare professional   Virtual or in-person, individual or group session with families in a similar situation to yours  Positive, negative  Yes or no |
| **D) Extra questions: future research, genetic testing, testing for other conditions**   1. *How do you feel about your child’s blood sample being stored for future research?*      1. *What if the screening test involved testing for genetics (DNA) of the child, to understand the child’s genetic risk of type 1 diabetes?*      1. *If a child was having a blood test to find out their risk of type 1 diabetes, would you want to have your child screened for other conditions they may have or be at risk of, like coeliac disease? We could do these tests on the same blood sample provided.*      1. *Can you think of any implications of knowing your child is at risk of a future condition?* | *The blood sample would be frozen. We would only perform additional tests with your consent. We would approach you for consent if this had not been given previously.* *Who could access the results?*  *This would involve either another blood test/more blood taking. Genetic risk factors for type 1 diabetes are an important area of research and provides additional information to antibody testing.*  *a. Would you prefer to have the antibody test / genetic test / both / or none?*  *b. Why?*  *What factors would your decision depend on?* | *Positive, negative*  *Positive, negative*  *Positive, negative*  *Positive, negative* |
| 1. Is there anything else that you would like to add? 2. Would you be interested in a follow-up interview?   Thank you for your time. | | |

**Table 1 legend:**

An outline of the topic guide used in the parents’ interviews. Questions and prompts marked in italic were optional and included if there was additional time available in the interview.
